# Machine learning for stroke in heart failure with reduced ejection fraction but without atrial fibrillation: A post‐hoc analysis of the WARCEF trial

**DOI:** 10.1111/eci.14360

**Published:** 2024-11-18

**Authors:** Hironori Ishiguchi, Yang Chen, Bi Huang, Ying Gue, Elon Correa, Shunichi Homma, John L. P. Thompson, Min Qian, Gregory Y. H. Lip, Azmil H. Abdul‐Rahim

**Affiliations:** ^1^ Liverpool Centre for Cardiovascular Science at University of Liverpool Liverpool John Moores University and Liverpool Heart & Chest Hospital Liverpool UK; ^2^ Division of Cardiology, Department of Medicine and Clinical Science Yamaguchi University Graduate School of Medicine Ube Japan; ^3^ Department of Cardiovascular and Metabolic Medicine, Institute of Life Course and Medical Sciences University of Liverpool Liverpool UK; ^4^ School of Computer Science and Mathematics Liverpool John Moores University Liverpool UK; ^5^ Columbia University Medical Center New York USA; ^6^ Danish Centre for Health Services Research, Department of Clinical Medicine Aalborg University Aalborg Denmark; ^7^ Stroke Division, Department Medicine for Older People Mersey and West Lancashire Teaching Hospitals NHS Trust Prescot UK

**Keywords:** heart failure with reduced ejection fraction, machine learning, stroke

## Abstract

**Background:**

The prediction of ischaemic stroke in patients with heart failure with reduced ejection fraction (HFrEF) but without atrial fibrillation (AF) remains challenging. Our aim was to evaluate the performance of machine learning (ML) in identifying the development of ischaemic stroke in this population.

**Methods:**

We performed a post‐hoc analysis of the WARCEF trial, only including patients without a history of AF. We evaluated the performance of 9 ML models for identifying incident stroke using metrics including area under the curve (AUC) and decision curve analysis. The importance of each feature used in the model was ranked by SAPley Additive exPlanations (SHAP) values.

**Results:**

We included 2213 patients with HFrEF but without AF (mean age 58 ± 11 years; 80% male). Of these, 74 (3.3%) had an ischaemic stroke in sinus rhythm during a mean follow‐up of 3.3 ± 1.8 years. Out of the 29 patient‐demographics variables, 12 were selected for the ML training. Almost all ML models demonstrated high AUC values, outperforming the CHA_2_DS_2_‐VASc score (AUC: 0.643, 95% confidence interval [CI]: 0.512–0.767). The Support Vector Machine (SVM) and XGBoost models achieved the highest AUCs, with 0.874 (95% CI: 0.769–0.959) and 0.873 (95% CI: 0.783–0.953), respectively. The SVM and LightGBM consistently provided significant net clinical benefits. Key features consistently identified across these models were creatinine clearance (CrCl), blood urea nitrogen (BUN) and warfarin use.

**Conclusions:**

Machine‐learning models can be useful in identifying incident ischaemic strokes in patients with HFrEF but without AF. CrCl, BUN and warfarin use were the key features.

## Graphical abstract

AF, atrial fibrillation; AUC, area under the curve; BUN, blood urea nitrogen; CrCl, creatinine clearance; DCA, decision curve analysis; DT, decision tree; GBM, gradient boosting machine; KNN, K‐nearest neighbours; LR, logistic regression; LVEF, left ventricular ejection fraction; MLP, multi‐layer perceptron; RF, random forest; SVM, support vector machine.

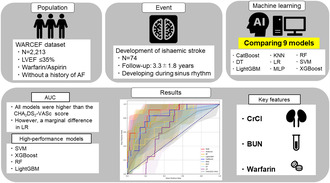

## 1 INTRODUCTION

The global prevalence of heart failure (HF), particularly in developed countries, is increasing, largely attributable to an ageing population.^1^, ^2^ Despite advancement in treatments, heart failure with reduced ejection fraction (HFrEF) remains a significant concern, often leading to poor outcomes.^3^ Ischaemic stroke profoundly affects patients with HF, who face a higher risk compared to individuals without AF, leading to worsening prognoses and increased hospitalisation costs.^4^

Atrial fibrillation (AF) is a significant contributor to ischaemic stroke in patients with HF, yet an elevated stroke risk persists even in those in sinus rhythm, particularly in cases of HFrEF.^5^ Clinical trials have primarily focused on assessing the effectiveness of prophylactic oral anticoagulants in HFrEF patients are in sinus rhythm,^6^, ^7^, ^8^, ^9^ but identification of high risk patients with HFrEF where anticoagulation shows a net clinical benefit remains suboptimal.

For example, the Warfarin versus Aspirin in Reduced Cardiac Ejection Fraction (WARCEF) Trial, a pivotal randomised controlled trial (RCT), examined the incidence of clinical events—a composite of death, ischaemic stroke and bleeding—in patients with HFrEF treated with either warfarin or aspirin.^6^ Although the primary endpoint of the trial showed no significant differences between the two groups, secondary analyses showed a significant reduction in ischaemic stroke incidence among patients treated with warfarin, but at the cost of higher major bleeding.^6^ Post‐hoc analysis of WARCEF study identified that factors such as prior stroke, left ventricular ejection fraction (LVEF), resting heart rate (HR) and time in therapeutic range (TTR) among warfarin‐treated patients significantly influenced the occurrence of ischaemic events.^10^, ^11^, ^12^ However, the accuracy of these factors in predicting ischaemic stroke risk during sinus rhythm remains uncertain. This uncertainty arises partly because the WARCEF trial cohort included approximately 13% of patients who either had some history of AF or developed AF during the follow‐up.

Machine learning (ML) has emerged as a promising method in contemporary clinical research, rapidly gaining prevalence. Prior studies demonstrated the ability of ML to identify patients developing AF during follow‐up.^13^, ^14^ However, research on the ML prediction of ischaemic stroke in patients with HFrEF but without AF remains limited. We aimed to assess the performance of ML to identify risk factors for ischaemic stroke in patients with HFrEF in sinus rhythm.

## 2 METHODS

### 2.1 Study design

We conducted a ML‐driven analyses to identify risk factors for ischaemic stroke incident utilising the data from the WARCEF trial. The conceptual framework and primary outcomes of this trial have been published elsewhere.^6^ Briefly, the trial was a 1:1 RCT that compared the efficacy of warfarin with aspirin, in patients with HFrEF, characterised by a LVEF of 35% or less, who were in sinus rhythm. The trial was conducted between October 2002 to January 2010, recruited a total of 2305 patients across 168 centres in 11 countries, with follow‐up periods lasting 6 years (mean duration 3.5 ± 1.8 years).

The WARCEF study was conducted in adherence to the ethical guidelines stipulated in the Declaration of Helsinki and received approval from the Institutional Review Boards and ethics committees of the coordinating centres at all participating sites. This post‐hoc analysis utilised anonymised WARCEF datasets.

### 2.2 Study endpoint

We selected patients who did not have a history of AF, with demographic data missing in less than 50% of cases, as our study population. The incidence of ischaemic stroke during sinus rhythm was set as the study endpoint. We excluded ischaemic stroke events that occurred after the development of AF in patients who developed AF during the follow‐up period.

### 2.3 Data pre‐processing

We analysed data on patient baseline demographics, including variables previously identified as critical factors for ischaemic stroke in prior post‐hoc analyses. These variables included LVEF, history of stroke and resting HR.^10^, ^11^, ^15^ For continuous variables, outliers were identified using a *Z*‐score threshold of greater than ±3 in a normal distribution. In non‐normal distributions, Tukey's outlier detection method was applied, identifying values less than 3 times the interquartile range (IQR) plus the first quartile or greater than 3 times the IQR plus the third quartile. The Miceforest programme was employed for the imputation of these outlier variables, along with other missing values, including categorical data.

From the analysed variables, we selected 12 features for inclusion into the ML models. This selection was guided by the application of the Boruta algorithm, information gain analysis and the Least Absolute Shrinkage and Selection Operator (LASSO) technique.^16^ To assess multicollinearity among the selected features, Pearson correlation coefficients (R‐values) and the Variance Inflation Factor (VIF) were calculated for each variable to evaluate multicollinearity. For categorical variables exhibiting *R*‐values of ≥0.7, one of each pair was omitted. When continuous variables with *R*‐values ≥0.7 or a VIF exceeding 10 were identified, one variable from each correlated pair was then binarised by its mean or median value to mitigate the effects of multicollinearity. If binarising the one variable did not sufficiently mitigate multicollinearity, the other variable in the pair was also binarised.

### 2.4 Machine learning

We compared the efficacy of 9 ML models in identifying patients who developed ischaemic stroke. These models included CatBoost, Decision Tree (DT), Light‐Gradient Boosting Machine (LightGBM), K‐Nearest Neighbours (KNN), Logistic Regression (LR), Multi‐Layer Perceptron (MLP), Random Forest (RF), Support Vector Machine (SVM) and XGBoost.

The cohort was partitioned into a training set (80%) and a test set (20%). Due to the imbalanced nature of the population, with a smaller proportion of patients with ischaemic stroke (74 out of 2213 patients; 3.3%), we employed the Synthetic Minority Over‐sampling Technique (SMOTE)^17^ for resampling the training cohort. Each model underwent training with adjusted hyperparameters, optimised through grid search results. To ensure the robustness and reliability of our findings, we conducted cross‐validations five times for the training cohort in each model. Upon establishing the optimal settings for each model, we evaluated the importance of each feature using Shapley Additive exPlanations (SHAP) values.^18^

### 2.5 Statistical analysis

Variables with normal distributions were represented as mean ± standard deviation, while those with non‐normal distributions were presented as medians with interquartile ranges (IQR; first and third quartiles). The Mann–Whitney U test was used to compare these variables. Categorical variables were expressed as numbers with percentages and compared using the Chi‐squared test. The performance of each ML algorithm was evaluated based on the area under the curve (AUC) with a 95% confidence interval (CI), alongside the optimal threshold determined by the Youden index applied across various metrics. The metrics involved precision (true positive number / total positive number), recall (sensitivity, true positive number/total ischaemic stroke number), F1 score (2 × [precision × recall]/[precision + recall]), accuracy ([true positive number + true negative number]/total number) and specificity. Receiver operator curves (ROC) were delineated for each model comparison. The CHA_2_DS_2_‐VASc score was included as a reference in the ROC analysis, using the entire cohort rather than the test cohort. Additionally, decision curve analysis (DCA) was also conducted to evaluate the net clinical benefit of each model. For the sensitivity analysis, we conducted ML analysis on a cohort superficially excluding individuals who had a history of AF as well as those who developed with incident AF during the study period. Results were presented statistically significant at a *p*‐value of less than .05. All statistical analyses were performed using R version 4.0.4. All programming tasks were executed using Python 3.10.12.

## 3 RESULTS

### 3.1 Study cohort

Of the total WARCEF cohort (*n* = 2305 randomised), we excluded 86 patients with prior history of AF, and an additional 6 with excessive (≥50%) missing data, leaving 2213 eligible for the current analysis (mean age 58 ± 11 years; 80% male). Among these, 215 (9.7%) patients developed AF during follow‐up. Additionally, 74 (3.3% of the 2213 initially eligible patients) developed ischaemic stroke during follow‐up (3.3 ± 1.8 years). In patients who developed AF, 13 events were documented after the development of AF. These events were not included as outcomes.

### 3.2 Patient demographics

Patient demographics are detailed in Table 1. Patients who developed ischaemic stroke during the study were significantly more likely to have a prior stroke compared to those who did not (26% vs. 10%, *p* < .001). The stroke incidence was lower in patients randomised to warfarin compared to patients randomised to aspirin. Furthermore, patients with ischaemic stroke had lower creatinine clearance (CrCl) and higher blood urea nitrogen (BUN) levels, although these differences did not reach statistical significance (*p* = .065 for CrCl and *p* = .101 for BUN).

**TABLE 1:** Patient demographics.

	Patients with ischaemic stroke (*n* = 74)	Patients without ischaemic stroke (*n* = 2139)	*p*‐value
Age (years), mean ± SD [0]	59 ± 12	58 ± 11	.328
Male, *n* (%) [0.2]	58 (78)	1713 (80)	.831
BMI (kg/m^2^), mean ± SD [0.5]	28 ± 5	29 ± 5	.171
White ethnicity, *n* (%) [0]	60 (81)	1718 (80)	.989
Live alone, *n* (%) [0]	18 (24)	474 (22)	.684
NYHA class >2, *n* (%) [0]	26 (35)	653 (31)	.473
LVEF (%), mean ± SD [6]	24 ± 7	25 ± 11	.705
Systolic BP (mmHg), mean ± SD [0]	124 ± 19	124 ± 18	.756
Diastolic BP (mmHg), mean ± SD [0]	74 ± 11	74 ± 11	.749
HR (/min), mean ± SD [0.1]	71 ± 13	72 ± 11	.543
Therapeutic agents			
Any antiplatelet agents, *n* (%) [0]*	49 (66)	1100 (51)	.017
Statin, *n* (%) [28]	61 (82)	1691 (79)	.577
ACEI/ARB, *n* (%) [0]	73 (99)	2108 (99)	>.999
BB, *n* (%) [0]	67 (91)	1924 (90)	>.999
CCB, *n* (%) [0.1]	4 (5)	189 (9)	.412
Diuretics, *n* (%) [0]	57 (77)	1725 (81)	.533
Laboratory findings			
CrCl (ml/min), mean ± SD [0.6]	78 ± 31	85 ± 32	.065
Haemoglobin (g/dl), mean ± SD [8]	14 ± 1	14 ± 1	.829
BUN (mg/dl), median (Q1, Q3) [3]	21 (17, 31)	20 (15, 27)	.101
Educational status [0.1]			.184
≤ 8th grade, *n* (%)	19 (26)	387 (18)	
Some high school, *n* (%)	10 (14)	550 (26)	
High school grad, *n* (%)	20 (27)	569 (27)	
Some colleges, *n* (%)	12 (16)	307 (14)	
College grad, *n* (%)	8 (11)	234 (11)	
Post‐grad education, *n* (%)	5 (7)	92 (4)	
Marital status [0]			.886
Married, *n* (%)	46 (62)	1366 (64)	
Single, *n* (%)	11 (15)	262 (12)	
Divorced, *n* (%)	8 (11)	276 (13)	
Widowed, *n* (%)	9 (12)	235 (11)	
Smoking [0.1]			.966
Current, *n* (%)	14 (19)	381 (18)	
Ex, *n* (%)	37 (50)	1100 (51)	
Never, *n* (%)	23 (31)	658 (31)	
Alcohol [0]			.456
Current, *n* (%)	14 (19)	541 (25)	
Ex, *n* (%)	18 (24)	467 (22)	
Never, *n* (%)	42 (57)	1131 (53)	
Comorbidities			
Hypertension, *n* (%) [3]	46 (62)	1305 (61)	.937
DM, *n* (%) [0.1]	28 (38)	662 (31)	.258
History of stroke, *n* (%) [0.1]*	19 (26)	220 (10)	<.001
Any vascular diseases, *n* (%) [0.2]	52 (70)	1325 (62)	.183
CAD, *n* (%) [0]	48 (65)	1280 (60)	.455
Agents			
Randomised to Aspirin/Warfarin [0]*			.016
Aspirin, *n* (%)	48 (65)	1069 (50)	
Warfarin, *n* (%)	26 (35)	1070 (50)	

*Note*: Numerical data are expressed as mean ± SD or median (interquartile range; first quartile, third quartile). Categorical data are expressed as numbers and percentages. Asterisk (*) indicates statistical significance (*p* < 0.05). [] indicates missing rate (%).

Abbreviations: ACEI, angiotensin‐converting enzyme inhibitor; ARB, angiotensin II receptor blocker; BB, beta blocker; BMI, body mass index; BP, blood pressure; BUN, blood urea nitrogen; CAD, coronary artery disease; CCB, calcium channel blocker; CrCl, creatinine clearance; DM, diabetes mellitus; HR, heart rate; LVEF, left ventricular ejection fraction; NYHA, New York Heart Association; SD, standard deviation.

### 3.3 Preprocessing variables

The missing rates for the variables were generally low (the median value of 0.1%). The highest missing rate was observed in the use of statin at 28%. A small proportion of values for systolic blood pressure (BP), heart rate (HR), creatinine clearance (CrCl) and blood urea nitrogen (BUN) were identified as outliers, accounting for 0.39%, 0.04%, 5% and 1% of the data, respectively. These variables were imputed using the MiceForest program.

### 3.4 Features selection

All the 29 variables encompassing patient demographics were organised according to their rankings as determined by Boruta algorithm (Figure S1A), the information gain (Figure S1B), and the LASSO techniques (Figure S1C). Among the variables that ranked high by any of these methods, systolic BP was excluded due to the highest VIF of 94, with a significant correlation with diastolic BP (*R* = 0.65). The use of antiplatelet agents was also excluded due to their high correlation with warfarin use (*R* = 0.95). Warfarin use was retained because of its relevance to the outcome. Remaining 12 variables were selected as features for the ML models. These variables include age, body mass index, diastolic BP, BUN, diabetes mellitus, CrCl, educational status, Haemoglobin, a history of stroke, HR, LVEF and warfarin use. Age, BMI, diastolic BP, HR, LVEF and haemoglobin levels were dichotomized based on their mean values due to high VIFs of 34, 42, 47, 37, 13 and 74, respectively. The final VIF values in these features are presented in Table S1.

### 3.5 Performance of each machine learning model

Table 2 details the performance metrics for each ML model. Table S2 shows the optimal hyperparameters identified through grid search.

**TABLE 2:** Comparison of machine learning models for identifying patients who developed ischaemic stroke.

	AUC	AUC: 95%CI	Optimal threshold	Precision	F1 Score	Accuracy	Sensitivity (recall)	Specificity
SVM	0.874	0.769–0.959	0.005	0.098	0.177	0.769	0.917	0.766
XGBoost	0.873	0.783–0.953	0.017	0.110	0.194	0.812	0.833	0.812
RF	0.843	0.725–0.942	0.185	0.186	0.291	0.911	0.667	0.919
LightGBM	0.828	0.705–0.941	0.002	0.157	0.253	0.893	0.667	0.900
CatBoost	0.823	0.694–0.927	0.187	0.089	0.161	0.767	0.833	0.763
KNN	0.819	0.674–0.934	0.097	0.084	0.154	0.752	0.833	0.749
MLP	0.741	0.584–0.883	0.0002	0.045	0.086	0.476	0.917	0.464
DT	0.735	0.582–0.879	0.125	0.114	0.192	0.867	0.583	0.875
LR	0.644	0.557–0.721	0.191	0.047	0.091	0.456	1.000	0.441

Abbreviations: AUC, area under the curve; CI, confidence interval; DT, decision tree; GBM, gradient boosting machine; KNN, K‐nearest neighbours; LR, logistic regression; MLP, multi‐layer perceptron; RF, random forest; SVM, support vector machine.

**FIGURE 1:**
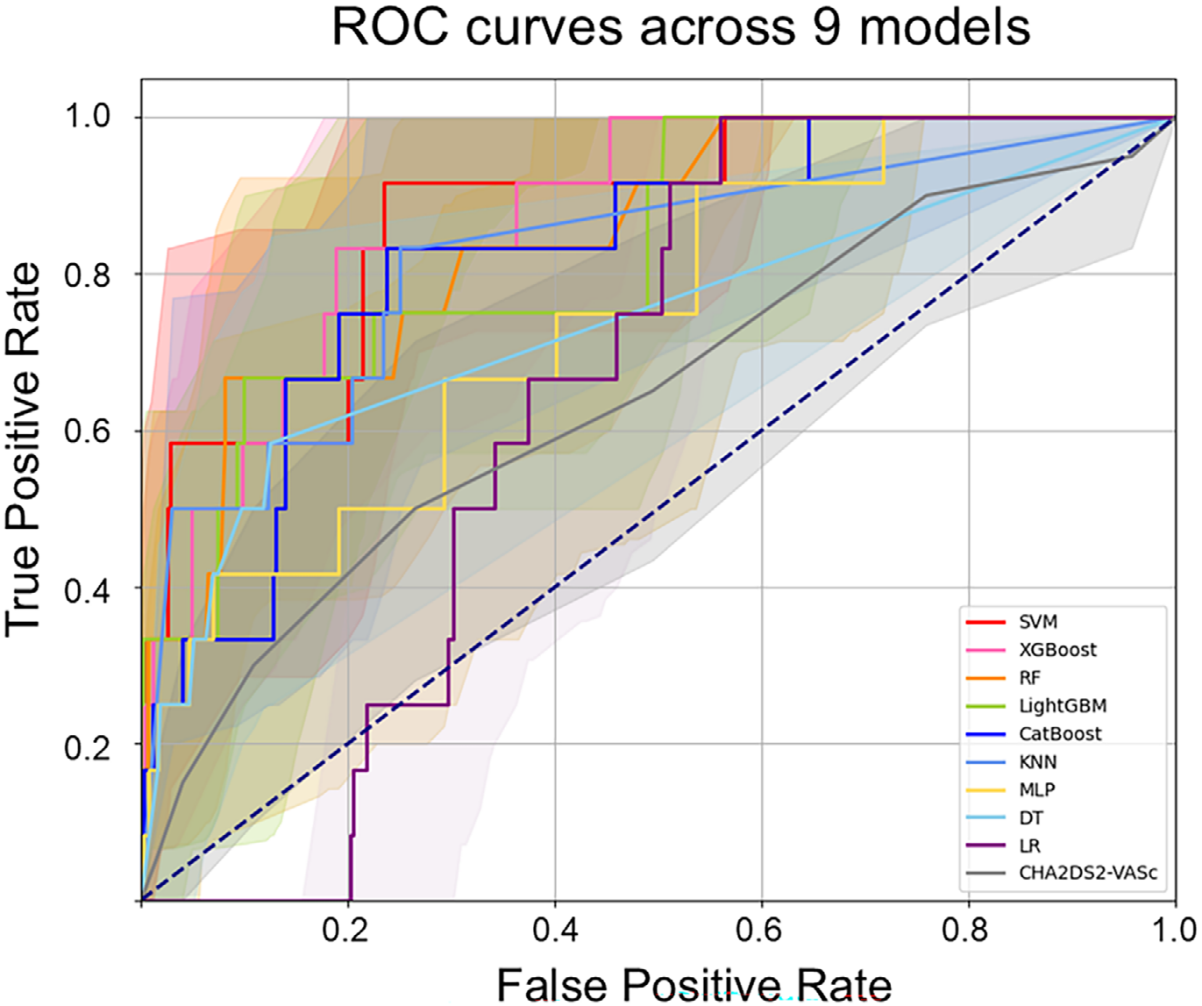
The comparison of the receiver operating characteristic curves for 9 models. The shaded area around each curve indicates the 95% CI. CI, confidence interval; DT, decision tree; GBM, gradient boosting machine; KNN, K‐nearest neighbours; LR, logistic regression; MLP, multi‐layer perceptron; RF, random forest; ROC, receiver operating characteristic; SVM, support vector machine.

All models in our study exhibited AUC values higher than those of the CHA_2_DS_2_‐VASc score (AUC 0.643, 95%CI: 0.512–0.767), although the improvement over this score was modest in the case of LR, as shown in Figure 1. The SVM model (AUC: 0.874, 95% CI: 0.769–0.959) and XGBoost model (0.873, 95% CI: 0.783–0.953) demonstrated high AUC values (≥0.85). Furthermore, the RF and LightGBM models exhibited high specificity (≥0.9).

**FIGURE 2:**
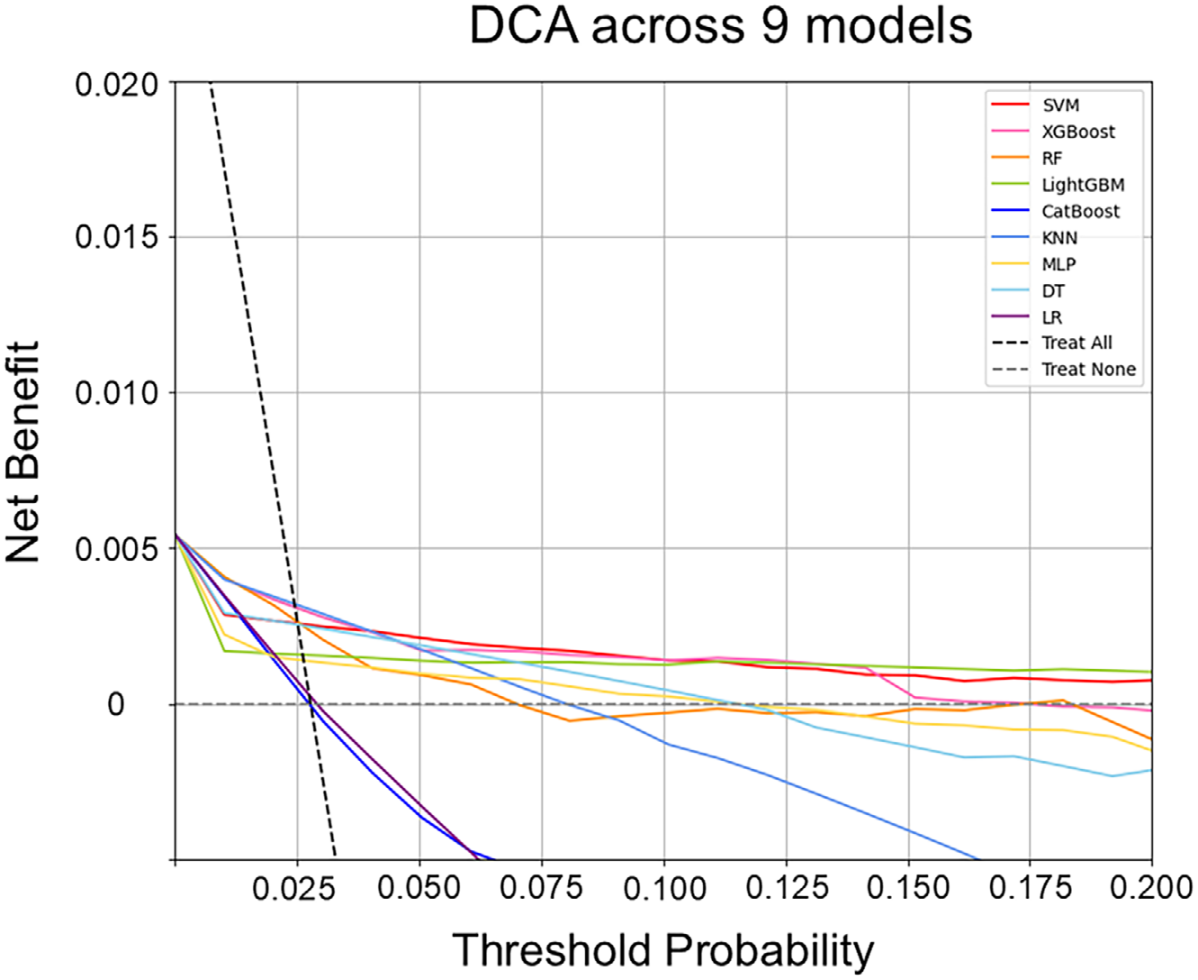
The comparison of the decision curve analysis for 9 models. DCA, decision curve analysis; DT, decision tree; GBM, gradient boosting machine; KNN, K‐nearest neighbours; LR, logistic regression; MLP, multi‐layer perceptron; RF, random forest; SVM, support vector machine.

DCA was performed to compare the net clinical benefit among the ML models (Figure 2). Except for LR model and CatBoost, all other models exhibited a higher net clinical benefit compared to the scenario of treating all patients. Remarkably, when the threshold probability was set below 0.2, both the SVM and LightGBM models consistently demonstrated a significant net clinical benefit. Similarly, the XGBoost model sustained a net clinical benefit at a threshold probability set below 0.15.

In the sensitivity analysis, we conducted analyses specifically excluding patients with either a history of AF or incident AF, comprising of 1998 subjects. The results from this refined cohort remained consistent with our initial findings (Table S3). The AUC exceeded 0.8 for all models, with the lone exception of the LR model. Additionally, the specificity exceeded 0.9 in all models except for the LR and DT models.

### 3.6 Important features

**FIGURE 3:**
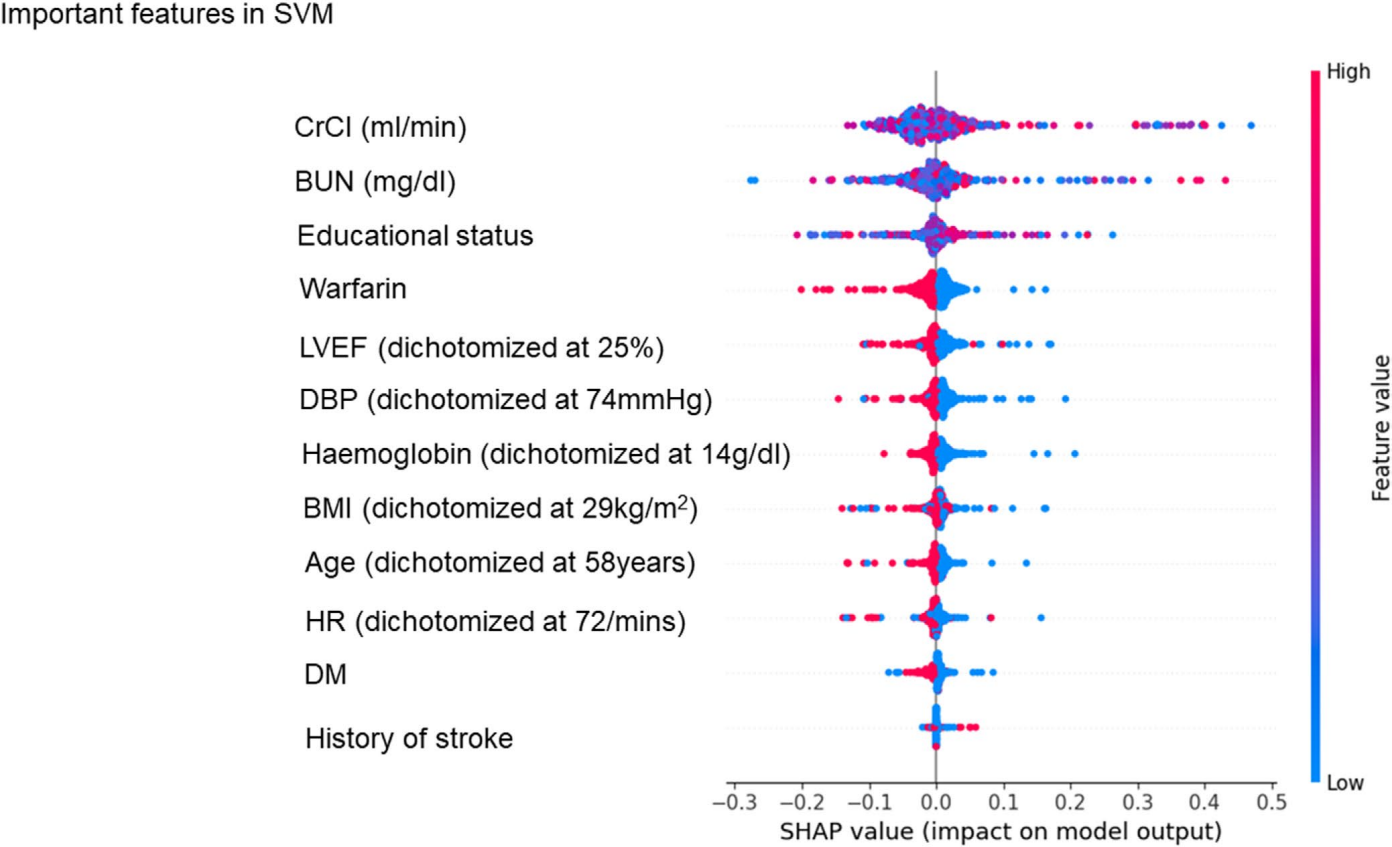
Feature rankings according to SHAP values in the Support Vector Machine model. Each feature is organised according to SHAP values. BMI, body mass index; BUN, blood urea nitrogen; CrCl, creatinine clearance; DBP, diastolic blood pressure; DM, diabetes mellitus; DT, decision tree; HR, heart rate; KNN, K‐nearest neighbours; LR, logistic regression; LVEF, left ventricular ejection fraction; MLP, multi‐layer perceptron; SHAP, SHapley Additive exPlanations.

**FIGURE 4:**
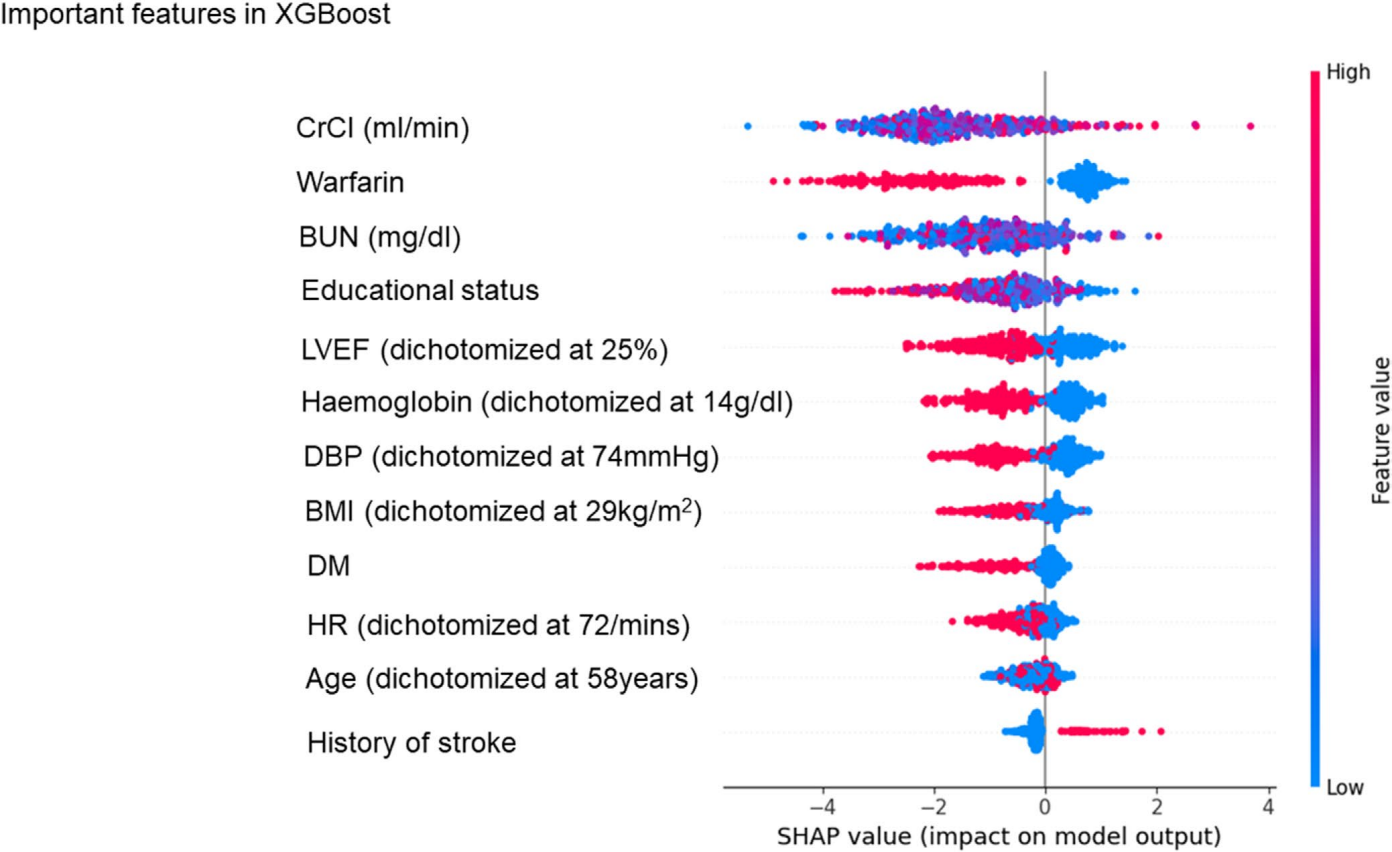
Feature rankings according to SHAP values in the XGBoost model. Each feature is organised according to SHAP values. BMI, body mass index; BUN, blood urea nitrogen; CrCl, creatinine clearance; DBP, diastolic blood pressure; DM, diabetes mellitus; HR, heart rate; LVEF, left ventricular ejection fraction; SHAP, SHapley Additive exPlanations.

**FIGURE 5:**
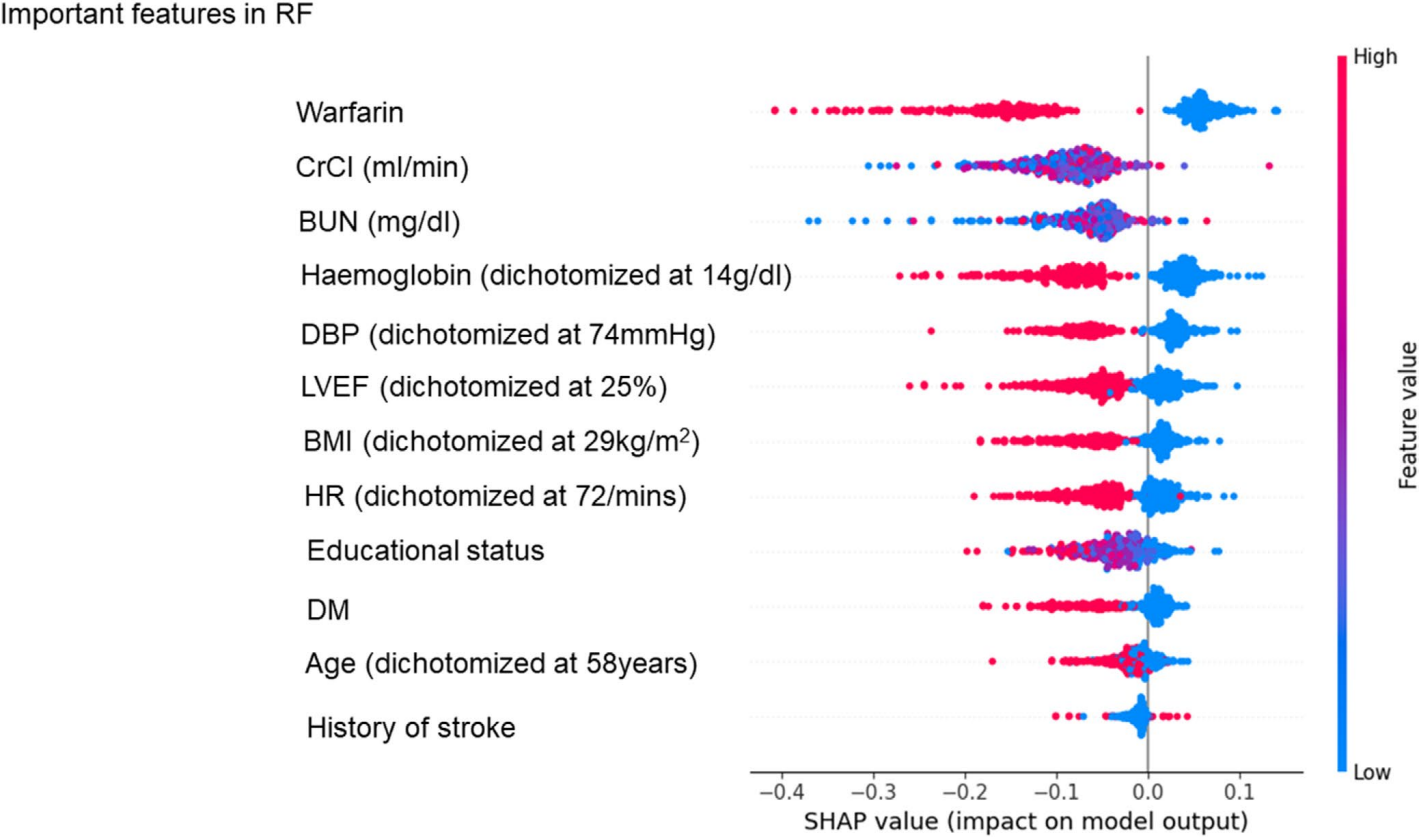
Feature rankings according to SHAP values in the Random Forest model. Each feature is organised according to SHAP values. BMI, body mass index; BUN, blood urea nitrogen; CrCl, creatinine clearance; DBP, diastolic blood pressure; DM, diabetes mellitus; HR, heart rate; LVEF, left ventricular ejection fraction; RF, random forest; SHAP, SHapley Additive exPlanations.

SHAP values were used to compare the contribution of features across each ML model, with the specific performance metrics for each model detailed in Figures 3, 4, 5 (SVM, XGBoost and RF, respectively) and Figure S2 (remaining 6 models). In high‐performance ML models with high AUC scores, CrCl and BUN consistently ranked as top 3 important features by SHAP values (Figures 3, 4, 5 and Figure S2A). The use of warfarin was also identified as an important feature for predicting the event, as it ranked the first in 5 out of 9 models.

## 4 DISCUSSION

The principal findings of this study are as follows (graphical abstract). First, all ML models demonstrated high AUC values, surpassing those of the CHA_2_DS_2_‐VASc score, in identifying patients who developed ischaemic stroke, within a cohort exclusively consisting of HFrEF patients in sinus rhythm, although with a marginal difference in LR. Second, the XGBoost, RF, SVM and LightGBM models showed high performances, achieving high scores in AUC. Third, SVM and LightGBM consistently provided significant net clinical benefits at a threshold probability of less than 0.2, using DCA. Finally, CrCl, BUN and warfarin usage emerged as primary key features for predicting ischaemic stroke.

### 4.1 Ischaemic stroke in heart failure

The mechanism of thromboembolism in patients with HFrEF is thought to be related to the fulfilment of ‘Virchow's triad’, that is abnormal blood stasis, abnormal blood constitutions and endothelial damage/dysfunction.^19^ Neurohormonal abnormalities and pro‐inflammatory pathways are implicated in promoting a hypercoagulable state and endothelial dysfunction. Blood stasis, primarily resulting from left ventricular dysfunction (e.g., left ventricular aneurysm), is recognised as a key factor in the occurrence of ischaemic stroke in patients with HFrEF.^19^ Left ventricular wall motion abnormalities are also often observed in patients who have thromboembolic strokes.^20^

Several trials, including the WARCEF trial, have demonstrated a reduction in ischaemic stroke incidence through the administration of oral anticoagulants in patients with HFrEF in sinus rhythm, but this is outweighed by the increased risk of major bleeding.^7^, ^8^, ^9^ Given the relatively low annual risk of ischaemic stroke in this patient population (approximately 1.0%), the indiscriminate use of anticoagulation therapy could lead to a situation where the associated risks exceed the potential benefits.^21^ Therefore, selecting patients where the benefits clearly outweigh the risks would be a key to make the most of benefit of this therapy.

### 4.2 Identifying high‐risk patients for ischaemic stroke in sinus rhythm

Several studies have aimed to identify the high‐risk populations for ischaemic stroke in HFrEF patients without AF.^22^, ^23^ For example, Abdul‐Rahim et al. identified predictors of stroke, including, age, higher New York Heart Association class, diabetes treated with insulin, decreasing body mass index, history of prior stroke and elevated levels of N‐terminal pro B‐type natriuretic peptide (NT‐proBNP), based on data from two major HF trials.^22^ They developed a scoring system derived from these variables, successfully distinguishing a high‐risk patient group with an annual stroke incidence of 2.2% and a *C*‐index of 0.75. Kondo et al. further refined this ischaemic stroke risk prediction model by focusing on history of prior stroke, diabetes treated with insulin and NT‐proBNP levels, using data from three contemporary HF trial cohorts.^23^ The refined model effectively identified a subset of high‐risk patients (2.1% annual stroke incidence) and achieved a *C*‐index of 0.84.

To the best of our knowledge, this study is the first to demonstrate the effectiveness of ML in identifying ischaemic stroke development within a cohort of HFrEF patients in sinus rhythm. All our ML models, with the exception of LR, exhibited AUC values ranging from 0.74 to 0.87, reaching levels comparable or better than those reported in previous studies.^22^, ^23^ The significant disparity in performance between LR and other ML models can likely be attributed to their varying abilities to handle non‐linear relationships. For example, Jang et al. previously showed that models proficient in non‐linear pattern recognition, such as SVM, RF and XGBoost, can surpass LR in predicting poor outcomes in ischaemic stroke patients.^24^ Our results are aligned with these observations.

### 4.3 Important features for identifying ischaemic stroke

In our study, CrCl and BUN were identified as key features, even after incorporating variables previously recognised as stroke risk factors in other studies.^10^, ^11^, ^22^, ^23^

The decline in renal function and the ensuing development of ischaemic stroke share common risk factors, including the promotion of atherosclerosis, with renal dysfunction significantly increasing the risk of stroke.^25^ In the chronic kidney disease (CKD) patient population, it has been reported that with each progressive stage of CKD increases the risk of stroke by approximately three to five times compared to the general population.^26^ This trend is more pronounced in the context of AF, as evidenced by previous research.^27^, ^28^

Recent meta‐analyses have demonstrated that patients with HF, irrespective of LVEF, exhibit a higher prevalence of CKD if they have experienced a stroke compared to those without a history of stroke.^29^ However, the debate continues regarding whether renal dysfunction serves as a direct risk factor for stroke or is merely a consequence of comorbidities such as hypertension and diabetes.^29^

In our findings, we propose that renal function may be a valuable indicator for identifying high‐risk patients for ischaemic stroke among those with HFrEF in sinus rhythm. Notably, our results suggest that BUN could be also a potential marker for high stroke risk, given that elevated BUN levels are indicative of dehydration and worsening renal function. We also identified the use of warfarin as a protective factor, aligned with prior analyses of the WARCEF trial,^6^, ^12^ but in our cohort which excluded individuals with a history of AF and censored for those who developed AF. Thus, our strengthens the evidence that warfarin use can offer protection against ischaemic stroke even in patients who are exclusively in sinus rhythm.

### 4.4 Clinical implications

Our study presents important clinical implications for managing patients with HFrEF in sinus rhythm. First, our ML models, particularly SVM, XGBoost, RF and LightGBM, demonstrated excellent performance in identifying patients at high risk of ischaemic stroke, despite its low overall incidence in this population. This approach could help clinicians to stratify risk more accurately than traditional methods. Secondly, our findings reinforce the protective role of warfarin against ischaemic stroke in HFrEF patients, even in the absence of AF. Notably, the emergence of renal function parameters such as CrCl and BUN, as key predictive features highlight the crucial role of kidney function in stroke risk assessment for HFrEF patients. This suggests that routine monitoring of renal function may play a vital role in stroke risk stratification, potentially identifying high‐risk patients who could benefit from anticoagulation therapy. Lastly, while ischaemic stroke is a relatively infrequent in HFrEF patients without AF, it remains an important and preventable complication. Some of our ML models demonstrated high accuracy in identifying high‐risk individuals, potentially enabling more targeted preventive strategies in this vulnerable population.

### 4.5 Limitations

However, our work has several limitations. First, external validation was not performed for our models, which raises uncertainties about their performance and generalisability in different populations. Particularly, most ischaemic stroke cases in our cohort were of the thromboembolic subtype, in contrast to a potentially higher proportion of atherosclerotic subtype cases in the general population.^15^ The variation of stroke subtypes could affect the generalisability of our findings. Second, our study did not include ML analysis of other completing outcomes, such as bleeding events and all‐cause mortality. To clarify, our focus on ischemic stroke, rather than all‐cause mortality—which is significantly more prevalent—was aimed to prevent the overshadowing of ischaemic stroke assessment by the sheer volume of mortality cases. Hence, our analysis of the net clinical benefit focused on the context of a ML metric, disregarding the broader implications of all‐cause mortality and its complex interplay with other outcomes. Third, we focused our analysis exclusively on the HFrEF population, which has significantly higher risks of both stroke and mortality compared to non‐HF groups. Although we identified renal function as an important feature, it remains unclear whether this applies to non‐HF populations as well. Forth, although we designated CHA_2_DS_2_‐VASc score as the reference, its effectiveness in estimating stroke risk in patients with sinus rhythm remains uncertain. However, previous literature suggests that the CHA_2_DS_2_‐VASc score may be equally effective in predicting stroke events in patients in sinus rhythm as in those with AF.^5^ Fifth, we utilised imputation for missing and outlier values. While this preprocessing has been employed in a previous ML post‐hoc analysis of WARCEF trial,^13^ it has not been commonly adopted in other studies. Although the frequency of these values is low, it may influence the generalizability. Sixth, while this study highlights the importance of CrCl and BUN levels in predicting ischaemic stroke, the practical implications of these findings—including whether baseline measurement is sufficient or if repeated measurements are necessary—still need to be determined through future research. This will help clarify how best to utilise these biomarkers in clinical practice to effectively manage and potentially prevent stroke this patient population. Finally, we defined the development of ischaemic stroke as a simple binary outcome, which is common in ML approaches. However, we acknowledge the importance of time‐to‐event data. Future research would benefit from advancements in ML techniques that effectively incorporate time‐to‐event information for more accurate prediction.

## 5 CONCLUSIONS

Machine learning models can identify risk factors for ischaemic stroke in patients with heart failure with reduced ejection fraction but without atrial fibrillation. Creatinine clearance, blood urea nitrogen and warfarin use were the key features to predict the ischaemic stroke.

## AUTHOR CONTRIBUTIONS

Conceptualization: H.I., E.C., A.H.A.R., G.Y.H.L. Data curation: S.H., J.L.P.T., G.Y.H.L. Formal analysis: H.I., Y.C. Investigation: H.I., Y.C., A.H.A.R. Methodology: H.I., A.H.A.R., G.Y.H.L. Project administration: H.I., A.H.A.R., G.Y.H.L. Resources: G.Y.H.L. A.H.A.R. Software: H.I. Visualisation: H.I., Y.C. Validation: H.I., Y.C. Writing‐orgininal draft: H.I. Writing‐review and editing: all authors. Supervision: G.Y.H.L., A.H.A.R.

## FUNDING INFORMATION

This study did not receive any specific grants from public, commercial, or non‐profit funding agencies.

## CONFLICT OF INTEREST STATEMENT

HI, BH, EC, SH, JLPT, MQ, AHAR report no conflicts of interest. GYHL reports Consultant and speaker for BMS/Pfizer, Boehringer Ingelheim, Daiichi‐Sankyo, Anthos. No fees are received personally. He is a National Institute for Health and Care Research (NIHR) Senior Investigator and co‐PI of the AFFIRMO project on multimorbidity in AF (grant agreement No 899871), TARGET project on digital twins for personalised management of atrial fibrillation and stroke (grant agreement no 101136244) and ARISTOTELES project on artificial intelligence for management of chronic long term conditions (grant agreement No 101080189), which are all funded by the EU's Horizon Europe Research & Innovation programme.

## Supporting information


Appendix S1.
