# Supplementary material for: Machine learning for stroke in heart failure with reduced ejection fraction but without atrial fibrillation: A post‐hoc analysis of the WARCEF trial
Source: Eur J Clin Invest. 2024 Nov 18;55(3):e14360. doi: 10.1111/eci.14360 (PMC11810539; doi:10.1111/eci.14360)
Supplement: Supplementary file 1 — Appendix S1. [file ECI-55-e14360-s001.docx]

**Supplementary-Figure 1. Feature Rankings from Three Different Algorithms**

(A) Boruta algorithm


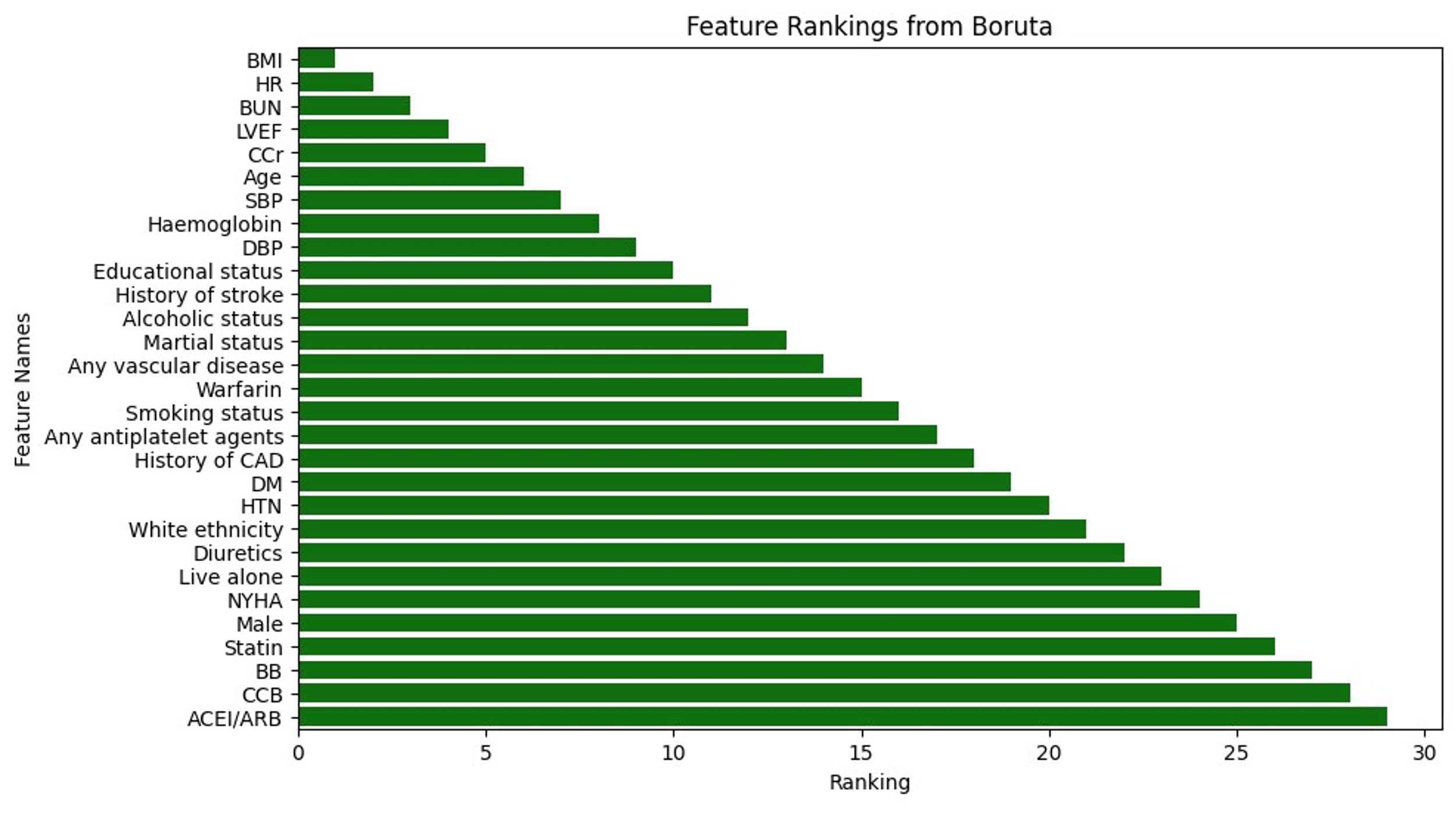


(B) Information gain


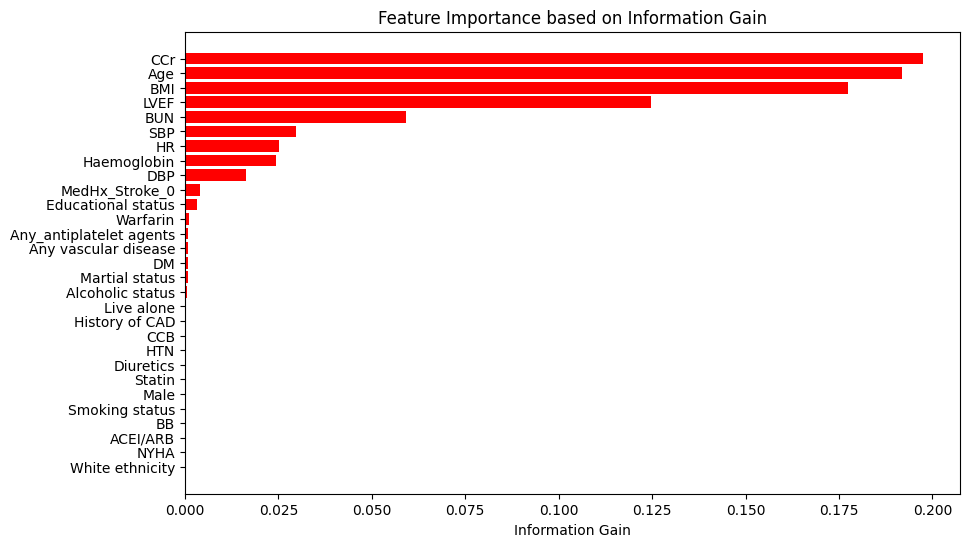


(C) LASSO technique


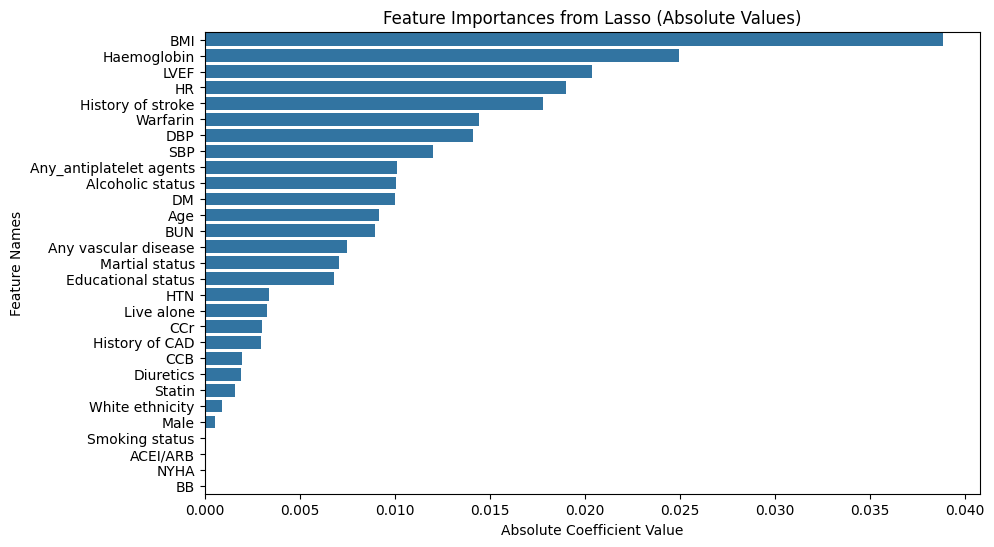


(A) The result of the Boruta algorithm. (B) The result of the information gain analysis. (C) The result of LASSO technique. E Each feature is organised according to its ranking. ACEI, angiotensin-converting enzyme inhibitor; ARB, angiotensin II receptor blocker; BB, beta blocker; BMI, body mass index; BP, blood pressure; BUN, blood urea nitrogen; CAD, coronary artery disease; CCB, calcium channel blocker; CCr, creatinine clearance; DM, diabetes mellitus; HR, heart rate; LASSO, Least Absolute Shrinkage and Selection Operator; LVEF, left ventricular ejection fraction; NYHA, New York Heart Association

**Supplementary-Figure 2. Feature Rankings According to SHAP Values in Each Model**

(A) Light GBM


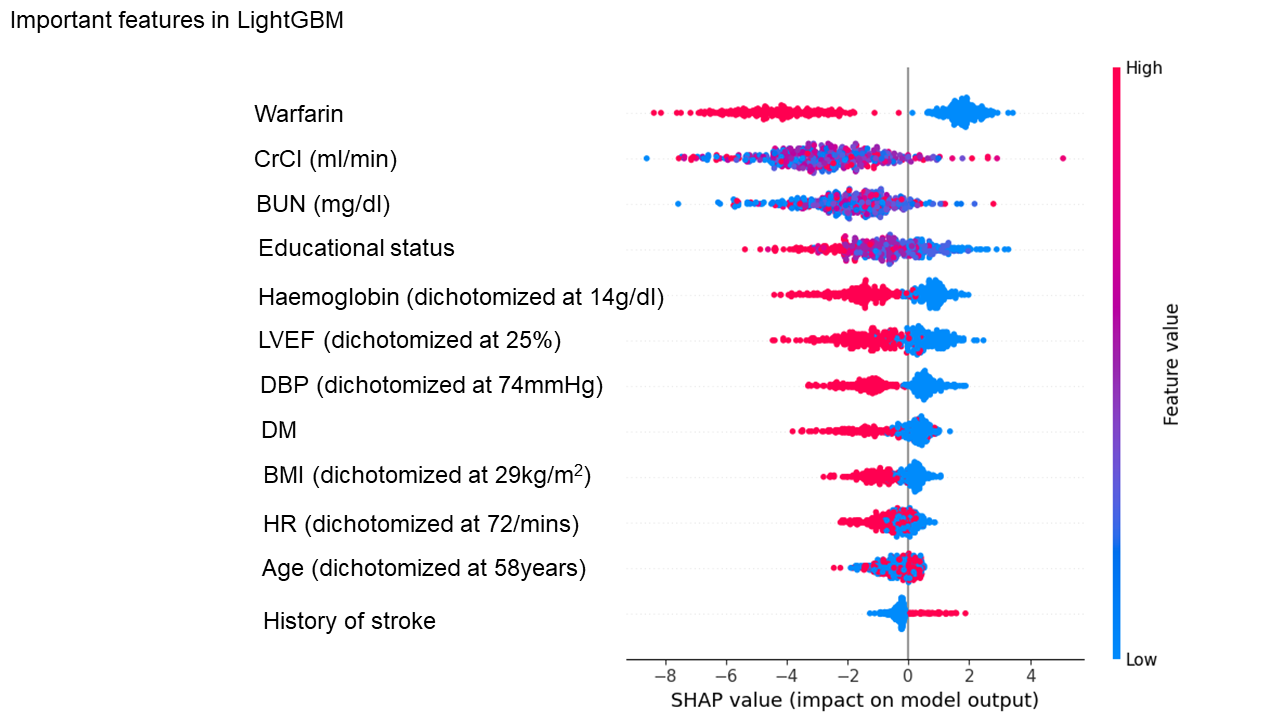


(B) CatBoost


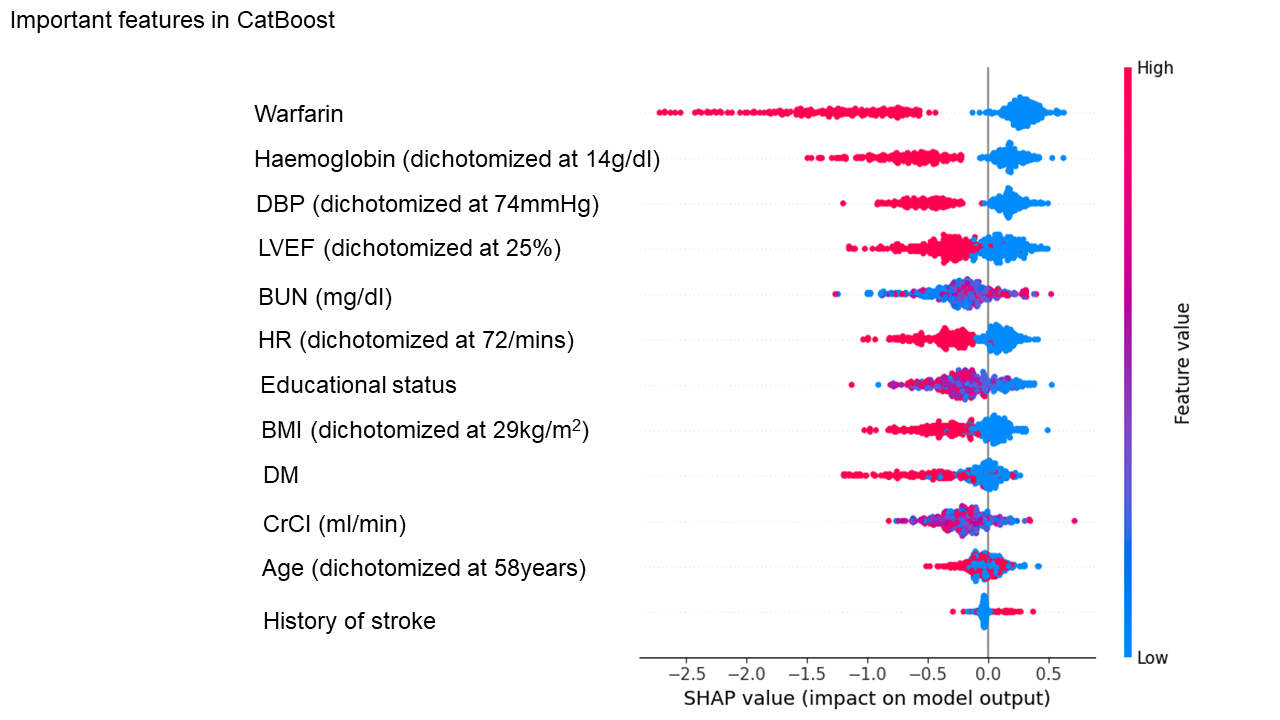


(C)KNN


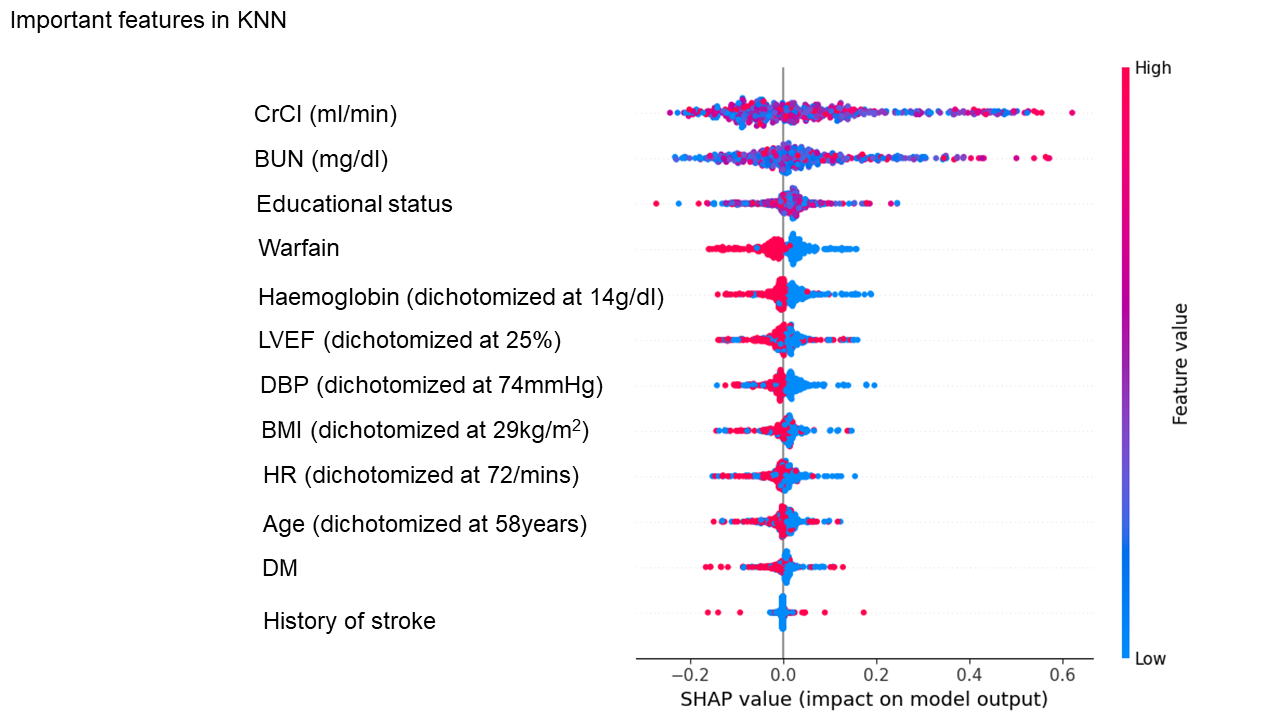


(D) MLP


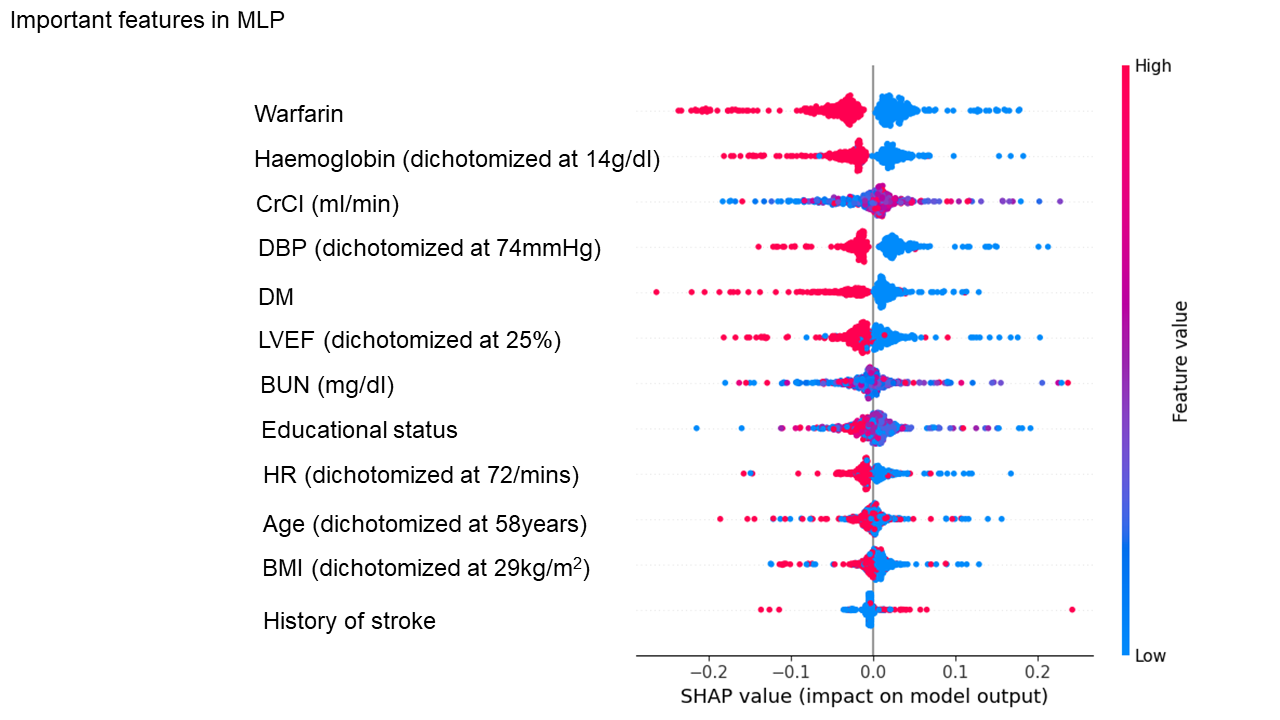


(E) DT


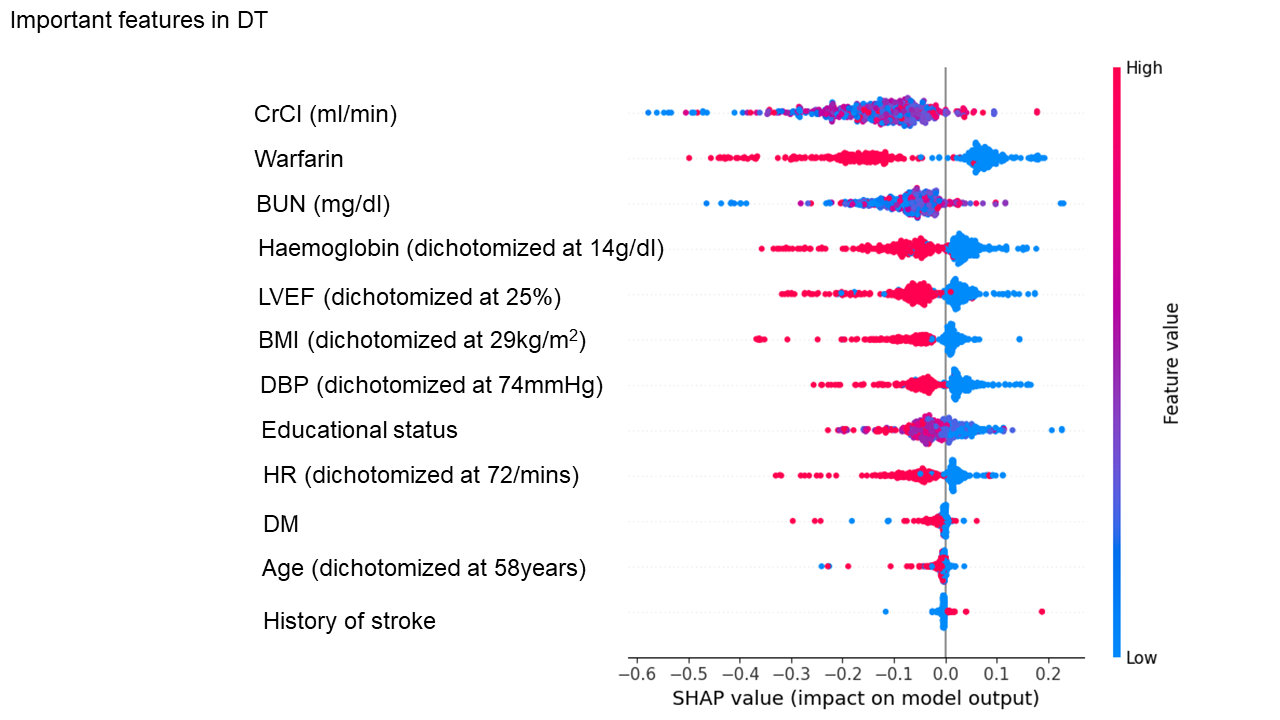


(F) LR


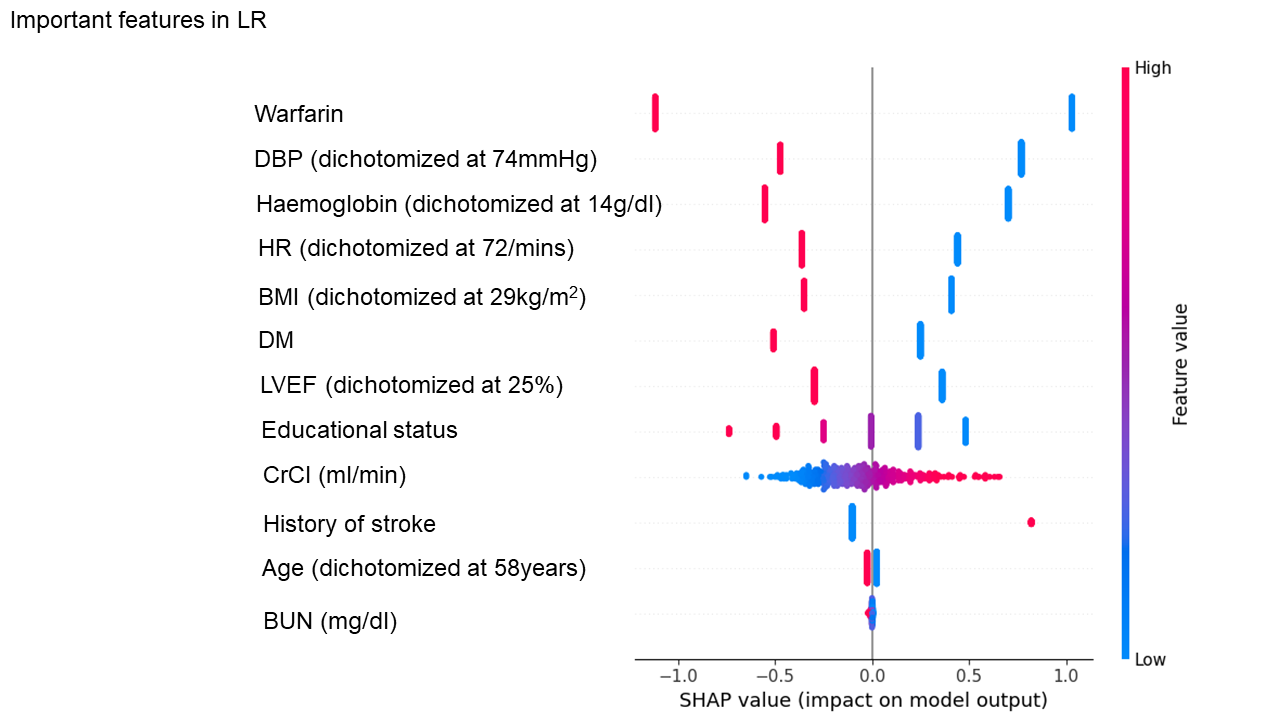


(A) Light GBM. (B) CatBoost. (C) KNN. (D) MLP. (E) DT. (F) LR. Each feature is organised according to SHAP values. BMI, body mass index; BUN, blood urea nitrogen; CrCl, creatinine clearance; DBP, diastolic blood pressure; DM, diabetes mellitus; DT, decision tree; GBM, gradient boosting machine; HR, heart rate; KNN, K-nearest neighbours; LR, logistic regression; LVEF, left ventricular ejection fraction; MLP, multi-layer perceptron; SHAP, SHapley Additive exPlanations

**Supplementary-Table 1. Variance Inflation Factor of Features Used in Machine Learning**

| Feature | VIF |
| --- | --- |
| Age (dichotomised by years) | 2.07 |
| BMI (dichotomised by 29 kg/m^2^) | 2.23 |
| DBP (dichotomized by 74mmHg) | 2.04 |
| HR (dichotomized by 72/mins) | 2.01 |
| LVEF (dichotomized by 25%) | 2.08 |
| Educational status | 2.66 |
| DM | 1.58 |
| History of stroke | 1.14 |
| Warfarin | 1.91 |
| CrCl (ml/min) | 7.06 |
| Haemoglobin (dichotomized by 14g/dl) | 2.25 |
| BUN (mg/dl) | 2.82 |

BMI, body mass index; BUN, blood urea nitrogen; CrCl, creatinine clearance; DBP, diastolic blood pressure; DM, diabetes mellitus; HR, heart rate; LVEF, left ventricular ejection fraction.

**Supplementary-Table 2. Hyperparameter Values in Each Model**

| Model | Hyperparameter | Value |
| --- | --- | --- |
| XGBoost | N estimators | 100 |
|  | Max depth | 6 |
|  | Learning rate | 0.3 |
|  | Reg lambda | 1 |
|  | Booster | gbtree |
| RF | N estimators | 200 |
|  | Max depth | None |
|  | Min samples split | 2 |
|  | Min samples leaf | 1 |
| SVM | C | 100 |
|  | Kernel | rbf |
|  | Degree | 4 |
|  | Gamma | auto |
| LightGBM | N estimators | 200 |
|  | Num leaves | 127 |
|  | Max depth | 10 |
|  | Learning rate | 0.1 |
|  | Subsample | 0.8 |
| CatBoost | Iteration | 50 |
|  | Depth | 10 |
|  | Learning rate | 0.1 |
|  | 12 leaf reg | 1 |
|  | Border count | 256 |
| KNN | N neighbours | 9 |
|  | Weights | distance |
|  | Algorithm | Auto |
|  | Leaf size | 10 |
|  | P | 1 |
| MLP | Hidden layer sizes | (100,50) |
|  | Activation | tanh |
|  | Solver | adam |
|  | Alpha | 0.001 |
|  | Learning rate | constant |
| DT | Max depth | None |
|  | Min samples split | 10 |
|  | Min samples leaf | 4 |
|  | max features | None |
| LR | C | 100 |
|  | Penalty | L2 |
|  | Solver | bilinear |

DT, decision tree; GBM, gradient boosting machine; KNN, K-nearest neighbours; LR, logistic regression; MLP, multi-layer perceptron; RF, random forest; SVM, support vector machine

**Supplementary-Table 3. Comparison of Machine Learning Model Performances for Identifying Patients who Developed Ischaemic Stroke (Sensitivity Analysis)**

|  | AUC | AUC: 95%CI | Optimal threshold | Precision | F1 Score | Accuracy | Sensitivity (recall) | Specificity |
| --- | --- | --- | --- | --- | --- | --- | --- | --- |
| SVM | 0.836 | 0.683-0.963 | 0.991 | 1.000 | 0.842 | 0.985 | 0.727 | 1.000 |
| XGBoost | 0.835 | 0.690-0.958 | 0.203 | 0.708 | 0.739 | 0.970 | 0.772 | 0.981 |
| RF | 0.914 | 0.824-0.983 | 0.335 | 0.708 | 0.739 | 0.970 | 0.772 | 0.981 |
| LightGBM | 0.868 | 0.733-0.980 | 0.421 | 0.944 | 0.850 | 0.985 | 0.772 | 0.997 |
| CatBoost | 0.905 | 0.815-0.978 | 0.305 | 0.658 | 0.708 | 0.965 | 0.772 | 0.976 |
| KNN | 0.878 | 0.783-0.963 | 1.000 | 0.695 | 0.711 | 0.967 | 0.727 | 0.981 |
| MLP | 0.843 | 0.724-0.952 | 0.930 | 0.941 | 0.820 | 0.982 | 0.727 | 0.997 |
| DT | 0.842 | 0.745-0.935 | 0.166 | 0.278 | 0.409 | 0.877 | 0.772 | 0.883 |
| LR | 0.681 | 0.483-0.739 | 0.141 | 0.084 | 0.150 | 0.577 | 0.681 | 0.571 |

AUC, area under the curve; CI, confidence interval; DT, decision tree; GBM, gradient boosting machine; KNN, K-nearest neighbours; LR, logistic regression; MLP, multi-layer perceptron; RF, random forest; SVM, support vector machine.
